# Identification of G protein-coupled receptor 55 (GPR55) as a target of curcumin

**DOI:** 10.1038/s41538-021-00119-x

**Published:** 2022-01-14

**Authors:** Naoki Harada, Mai Okuyama, Yoshiaki Teraoka, Yumi Arahori, Yoh Shinmori, Hiroko Horiuchi, Paula B. Luis, Akil I. Joseph, Tomoya Kitakaze, Shigenobu Matsumura, Tohru Hira, Norio Yamamoto, Takashi Iuni, Naoki Goshima, Claus Schneider, Hiroshi Inui, Ryoichi Yamaji

**Affiliations:** 1 Division of Applied Life Sciences, Graduate School of Life and Environmental Sciences, Osaka Prefecture University, Sakai, Osaka, 599-8531 Japan; 2 Department of Pharmacology and the Vanderbilt Institute of Chemical Biology, Vanderbilt University Medical School, Nashville, TN 37232 USA; 3 Division of Clinical Nutrition, Graduate School of Comprehensive Rehabilitation, Osaka Prefecture University, Habikino, Osaka, 583-0872 Japan; 4 Research Faculty of Agriculture, Hokkaido University, Sapporo, Hokkaido 060-8589 Japan; 5 R&D Planning Division, House Wellness Foods Corporation, Yotsukaido, Chiba, 284-0033 Japan; 6 Molecular Profiling Research Center for Drug Discovery, National Institute of Advanced Industrial Science and Technology, Koto-ku, Tokyo, 135-0064 Japan; 7 Department of Human Sciences, Musashino University, Koto-ku, Tokyo, 135-8181 Japan; 8 Department of Health and Nutrition, Otemae University, Osaka, 540-0008 Japan

**Keywords:** Nutrition, Type 2 diabetes

## Abstract

The identification of molecular targets of bioactive food components is important to understand the mechanistic aspect of their physiological functions. Here, we have developed a screening system that enables us to determine the activation of G protein-coupled receptors (GPCRs) by food components and have identified GPR55 as a target for curcumin. Curcumin activated GPR55 and induced serum-response element- and serum-response factor-mediated transcription, which were inhibited by Rho kinase and GPR55 antagonists. Both the methoxy group and the heptadienone moiety of curcumin were required for GPR55 activation. The F190^5.47^ residue of GPR55 was important for the interaction with curcumin. The curcumin-induced secretion of glucagon-like peptide-1 in GLUTag cells was inhibited by a GPR55 antagonist. These results indicate that expression screening is a useful system to identify GPCRs as targets of food components and strongly suggest that curcumin activates GPR55 as an agonist, which is involved in the physiological function of curcumin.

## Introduction

Curcumin is the predominant bioactive curcuminoid in turmeric (*Curcuma longa*), which is widely used as a spice for preparing curry. Turmeric contains demethoxycurcumin and bisdemethoxycurcumin as minor curcuminoids^1^. Curcumin is absorbed by the body and is partly metabolized to tetrahydrocurcumin, ferulic acid, and vanillin^1^. Tetrahydrocurcumin, a major metabolite of curcumin, can also be produced by the gut microbiota^2^. Curcumin co-exists as the diketo and keto-enol tautomers in solution^3^. Various activities of curcumin, such as anti-diabetic, anti-oxidative, anti-tumorigenic, anti-inflammatory, and neurotrophic properties, have been widely investigated^4–6^. The anti-diabetic property of curcumin is due to the induction of glucagon-like peptide-1 (GLP-1) secretion in intestinal L-cells^7–9^. However, the molecular mechanisms by which curcumin exerts its biological effects have not yet been fully elucidated.

G protein-coupled receptors (GPCRs) are seven-transmembrane receptors that form the largest family of cell-surface receptors. Humans express approximately 800 genes encoding receptors in this family. GPCRs mediate cellular responses to various extracellular stimuli, such as ions, photons, lipids, peptides, proteins, and small molecules^10^. GPCRs are important targets of drugs due to their ability to initiate cellular signaling pathways and their selective expression in target cells^11^. These properties enable GPCRs to regulate diverse physiological processes selectively. Although small molecules, such as polyphenols, in food have been found to activate GPCRs^12–14^, the specific ligand–receptor combinations are poorly understood.

Heterotrimeric G proteins consisting of α, β, and γ subunits mediate GPCR activation and intracellular signal transduction. G proteins are classified into Gs, Gi/Go, Gq, and G_12/13_ subfamilies according to their α subunits^15^. The activation of GPCRs coupled to the Gs subunit activates cAMP/PKA signaling and induces cAMP-response element (CRE)-mediated transcription, which is countered by the activation of GPCRs coupled to the Gi/Go subunit. The activation of GPCRs coupled to Gi/Go, Gq, and G_12/13_ activates serum-response element (SRE)-mediated transcription. While Gi/Go and Gq stimulate Raf/MEK/ERK signaling, G_12/13_ stimulates RhoA/ROCK signaling, both of which activate SRE-mediated transcription. The activation of G_12/13_ also induces serum-response factor-response element (SRF-RE)-mediated transcription, whereas the activation of Gq stimulates nuclear factor of activated T-cell response element (NFAT-RE)-mediated transcription^10,11,15^.

Identifying the binding partners of food components is required to fully understand the mechanisms underlying their physiological functions and to explore the unknown physiological functions. In this study, we have identified G protein-coupled receptor 55 (GPR55) as a target of curcumin. Moreover, the potential role of GPR55 in the physiological function of curcumin was investigated.

## Results

### Activation of GPR55 by curcumin and determination of the associated G-protein

**Fig. 1:**
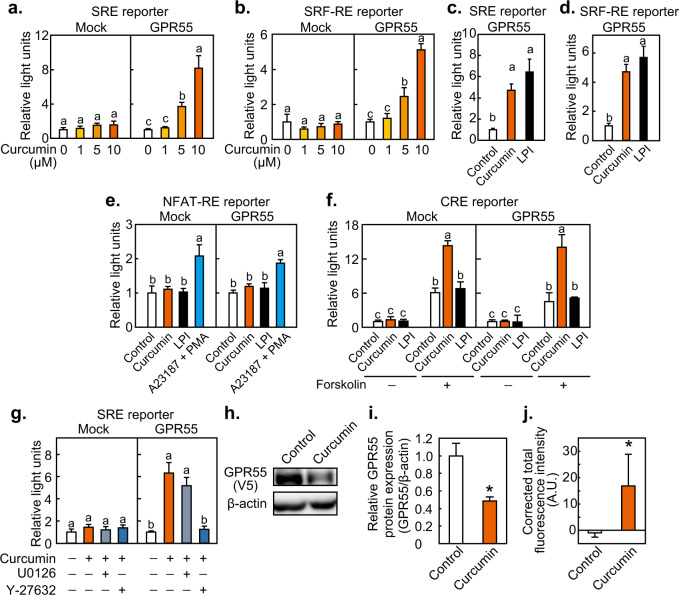
Activation of GPR55 by curcumin. HEK293-FT cells transfected with a GPR55 expression vector and firefly and *Renilla* luciferase reporter vectors were stimulated with curcumin or lysophosphatidylinositol (LPI), and reporter assay was performed. The effects of curcumin and LPI were determined on (**a**, **c**) serum reponse element (SRE)-mediated transcription, (**b**, **d**) serum-response factor-response element (SRF-RE)-mediated transcription, (**e**) nuclear factor of activated T-cells-response element (NFAT-RE)-mediated transcription, and (**f**) cAMP-response element (CRE)-mediated transcription. **g** Effects of curcumin and signal transduction pathway inhibitors on SRE-mediated transctiption. Cells were stimulated by the indicated concentrations or 10 µM curcumin, 300 nM LPI, 5 µM A23187 + 10 ng/ml phorbol myristate acetate (PMA), 10 µM forskolin, 10 µM U0126, and/or 10 µM Y-27632. (**h**, **i**) GPR55 protein levels after stimulation with 10 μM curcumin for 3 h. The band intensity of GPR55 was expressed relative to the band intensity of β-actin. **j** The GPR55-dependent change in Fura-8 fluorescence was determined by subtracting the fluorescence value in the vector-transfected cells from the fluorescence value in the GPR55-expressing cells. Data were expressed as the mean ± s.d. (*n* = 4) for luciferase and calcium assays or (*n* = 3) for western blotting. Statistical analysis was performed on each of the wells transfected with the mock vector or the GPR55 expression vector. Different letters or an asterisk indicate significant differences (*p* < 0.05).

To elucidate GPCRs as possible receptors for curcumin, we performed an expression screening. HEK293FT cells were transfected with GPCR expression vectors and a p4xCRE-3xSRE-TATA-Luc2P reporter vector followed by stimulation with curcumin. After screening 258 human GPCRs, we identified GPR55 as a potential target of curcumin, based on a significant activation of the reporter construct by curcumin. Further analysis showed that curcumin-induced SRE-mediated transcriptional activity (i.e., activation of Gi/Go, Gq, and/or G_12/13_) in a dose-dependent manner in cells expressing GPR55 (Fig. 1a). Curcumin also induced the SRF-RE-mediated transcriptional activity (i.e., activation of G_12/13_) in a GPR55-dependent manner (Fig. 1b). In both cases, the effects of curcumin were significant at concentrations above 5 µM. The enhancement in SRE- or SRF-mediated transcription by curcumin at 10 µM was similar to that achieved by 300 nM lysophosphatidylinositol (LPI), an endogenous ligand for GPR55 (Figs. 1c, d). In contrast, NFAT-RE- or CRE-mediated transcriptional activities (*i.e*., activation of Gq or Gs, respectively) were not stimulated by curcumin or LPI in a GPR55-dependent manner (Figs. 1e, f). Calcium ionophore A23187 and the protein kinase C activator phorbol myristate acetate (PMA) were used as positive control for validation of NFAT-RE-mediated transcriptional activities. Additionally, curcumin and LPI did not suppress CRE activity induced by the adenylate cyclase activator forskolin (*i.e*., activation of Gi), and curcumin activated CRE independent of GPR55 (Fig. 1f). The GPR55-independent and forskolin-dependent CRE activation by curcumin may be due to its phosphodiesterase inhibitory activity^16^. The SRE-mediated transcriptional activation by curcumin was suppressed by the ROCK inhibitor Y-27632, but not by the MEK1/2 inhibitor U0126 (Fig. 1g). The protein level of GPR55 was significantly decreased by curcumin (Figs. 1h, i, and Supplementary Fig. 1), consistent with receptor internalization and degradation upon ligand activation^17^. In addition, curcumin increased intracellular Ca^2+^ levels in a GPR55-dependent manner (Fig. 1j) further verifying the activation of GPR55 by curcumin. Collectively, these results indicated that curcumin activates GPR55 and induces G_12/13_, but not Gs, Gq, and Gi, signaling.

### Effects of curcumin-related compounds on GPR55 activation

**Fig. 2:**
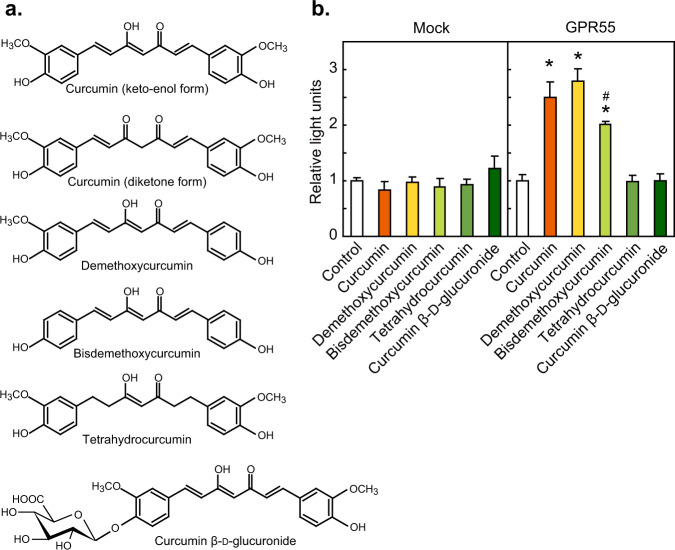
Effects of curcuminoids and tetrahydrocurcumin on the activation of GPR55. **a** Chemical structures of curcuminoids, the major curcumin metabolite tetrahydrocurcumin, and the major conjugate curcumin β-d-glucuronide. **b** HEK293-FT cells transfected with the GPR55 expression vector, p3xSRE-TATA-Luc2P, and pGL4.74[*hRluc*/TK] were stimulated with each compound at 10 µM and reporter assay was performed. Data were expressed as the mean ± s.d. (*n* = 4). Statistical analysis was performed on each of the wells transfected with the mock vector or the GPR55 expression vector. *p < 0.05 vs control; ^**#**^*p* < 0.05 vs^*.*^ both control and compound 1 (curcumin).

We compared the activation of GPR55 by curcumin with the minor curcuminoids demethoxycurcumin and bisdemethoxycurcumin, the reduced metabolite tetrahydrocurcumin, and the conjugate curcumin β-d-glucuronide, which are present in vivo upon consumption of turmeric containing foods (Fig. 2a). While demethoxycurcumin activated SRE-mediated transcription at a level similar to curcumin, the activation by bisdemethoxycurcumin was weaker, whereas tetrahydrocurcumin and curcumin β-d-glucuronide did not activate the receptor (Fig. 2b).

**Fig. 3:**
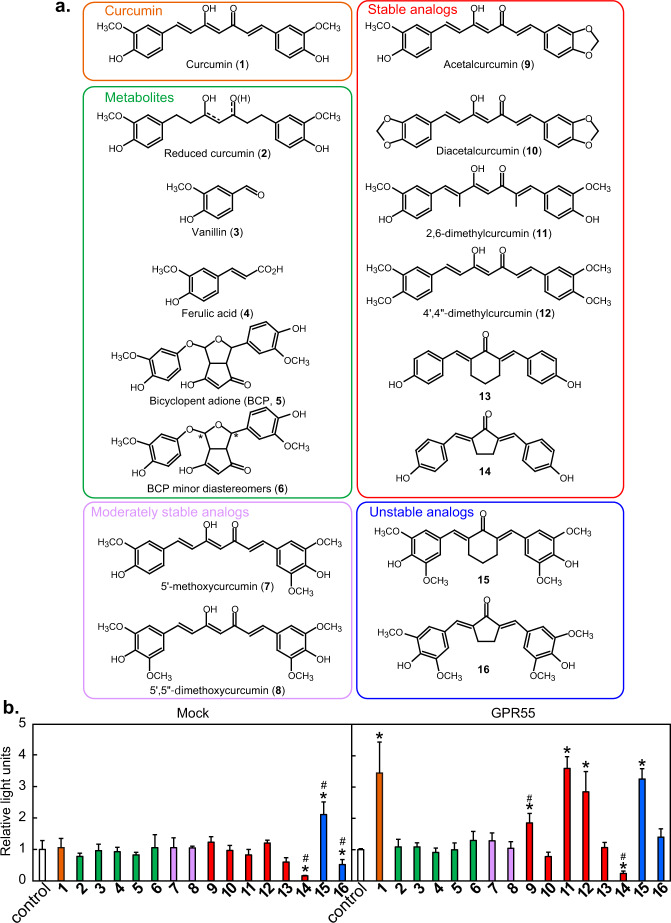
Structure–activity relationship of GPR55 activation by curcumin and curcumin-related compounds. **a** Chemical structures of curcumin metabolites, moderately stable analogs, stable analogs, and unstable analogs. **b** HEK293-FT cells transfected with the GPR55 expression vector, p3xSRE-TATA-Luc2P, and pGL4.74[*hRluc*/TK] were stimulated with each compound at 10 µM and reporter assay was performed. Data were expressed as the mean ± s.d. (*n* = 4). Statistical analysis was performed on each of the wells transfected with the mock vector or the GPR55 expression vector. *p < 0.05 vs control; ^**#**^*p* < 0.05 vs^*.*^ both control and compound 1 (curcumin).

Since curcumin can degrade through oxidation^18–21^ we analyzed its stability in our luciferase reporter assay conditions for 4 h and found that the curcumin-dependent OD_425_ value was decreased by 16% (data not shown). Then, we further evaluated the effects of its metabolites (reduced curcumin (**2**)^22^, vanillin (**3**), ferulic acid (**4**), bicyclopentadione (BCP, **5**), BCP minor diastereomer (**6**)), as well as moderately stable analogs (5′-methoxycurcumin (**7**), 5′,5′′-methoxycurcumin (**8**)), stable analogs (acetalcurcumin (**9**), diacetalcurcumin (**10**), 2,6-dimethylcurcumin (**11**), 4′,4′′-dimethylcurcumin (**12**), stable cyclohexanone derivatives of curcumin (**13**), and stable cyclopentanone derivatives of curcumin (**14**)) and unstable analogs (unstable cyclohexanone derivatives of curcumin (**15**), and unstable cyclopentanone derivatives of curcumin (**16**)) for GPR55 activation (Fig. 3a). The stability of the analogs is based on their sensitivity to autoxidation, and the moderately stable **7** and **8** degrade slower than curcumin whereas unstable **15** and **16** degrade faster^20^. None of the curcumin metabolites activated SRE-mediated transcription (Fig. 3b) while 2,6-dimethylcurcumin (**11**) and 4′,4′′-dimethylcurcumin (**12**) activated the transcription to levels similar to those as curcumin, and compound **9** (acetalcurcumin) marginally but significantly activated SRE-mediated transcription. Unexpectedly, compound **15** activated SRE-mediated transcription independent of GPR55, and thus should not be considered an activating compound. Testing of the metabolites and analogs indicated that curcumin was the major naturally occurring activator of GPR55 and that both the methoxy group and the heptadienone moiety of curcumin were required for the maximal effects.

### Effects of GPR55 antagonists on curcumin-induced GPR55 activation

**Fig. 4:**
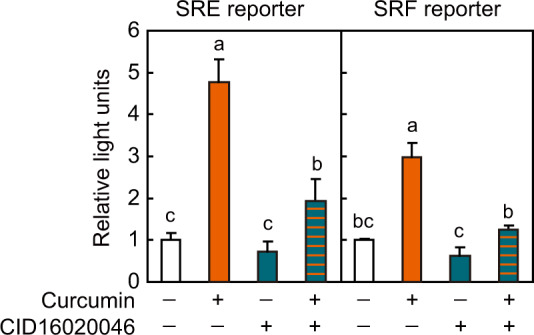
Effects of GPR55 antagonists on GPR55 activation. HEK293-FT cells transfected with the GPR55 expression vector, p3xSRE-TATA-Luc2P or p2xSRF-TATA-Luc2P, and pGL4.74[*hRluc*/TK] were stimulated with 10 µM curcumin for 4 h, and reporter assay was performed. Cells were preincubated with GPR55 antagonist (10 µM CID16020046) or vehicle for 30 min before curcumin treatment. Data were expressed as the mean ± s.d. (*n* = 4). Different letters indicate significant differences (*p* < 0.05).

To examine whether curcumin acts as an agonist for GPR55, the GPR55 antagonist CID16020046 was employed. As shown in Fig. 4, CID16020046 suppressed the induction of SRE- or SRF-RE-mediated transcription by curcumin. These results suggest that curcumin binds to the ligand-binding pocket of GPR55.

### Structure-based docking of curcumin to GPR55 and its validation

**Fig. 5:**
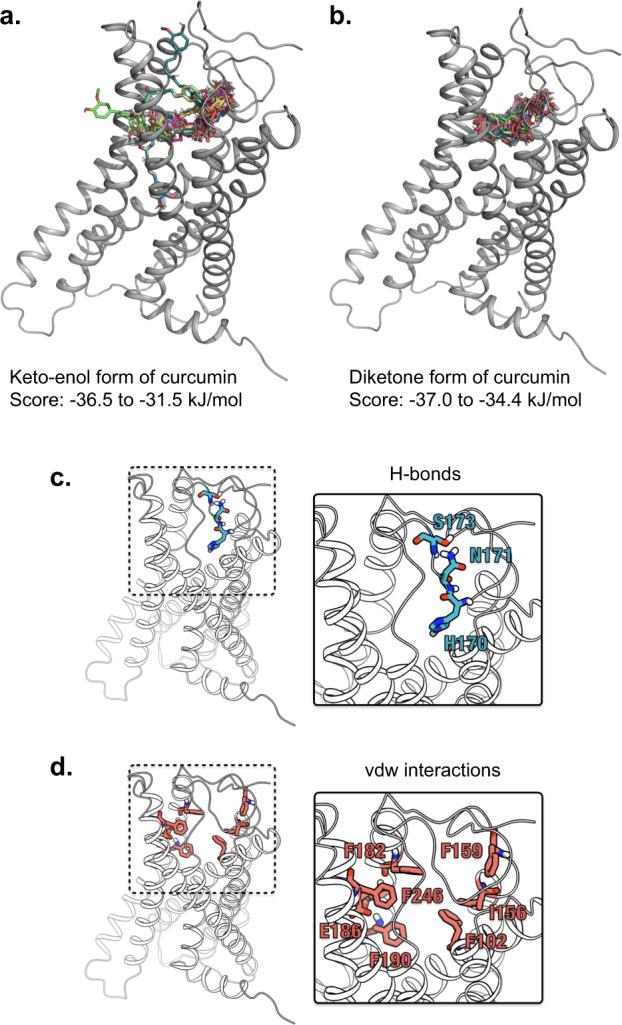
Docking simulation of curcumin with GPR55. Molecular docking between curcumin and GPR55 was performed with AutoDock Vina. **a**, **b** After 100 docking simulations using the keto-enol and diketone forms of curcumin, respectively, the estimated binding forms and binding free energies values are shown. **c**, **d** Key amino acids for binding to curcumin are predicted with AutoMap analyses. Amino acid residues involved in hydrogen bonds (**c**) and van der Waals interactions (**d**) between GPR55 and curcumin are indicated.

To identify key residues of GPR55 involved in curcumin binding, we computationally docked two tautomers of curcumin (i.e., enol and diketone forms; Fig. 2a) against the model structure of GPR55 using AutoDock Vina. Regardless of the tautomeric form of curcumin, most binding poses were located at the same sites of GPR55 (Figs. 5a, b), and the docking scores of the enol and diketone forms ranged from –36 to –31 kJ mol^–1^ and –37 to –34 kJ mol^–1^, respectively. We determined GPR55 residues that were frequently involved in interactions with the curcumin tautomers in all obtained poses because the differences in the docking score were insufficient for considering only the top-ranked pose. Three residues (H170^ECL2^, N171^ECL2^, and S173^5.31^) on the extracellular loops formed hydrogen bonds with high frequency (Fig. 5c). By contrast, one residue (F159^4.64^) on the extracellular loops and six residues (F102^3.33^, I156^4.60^, F182^5.40^, E186^5.44^, F190^5.47^, and F246^6.55^) on the transmembrane helices were involved in vdW interactions with high frequencies (Fig. 5d).

**Fig. 6:**
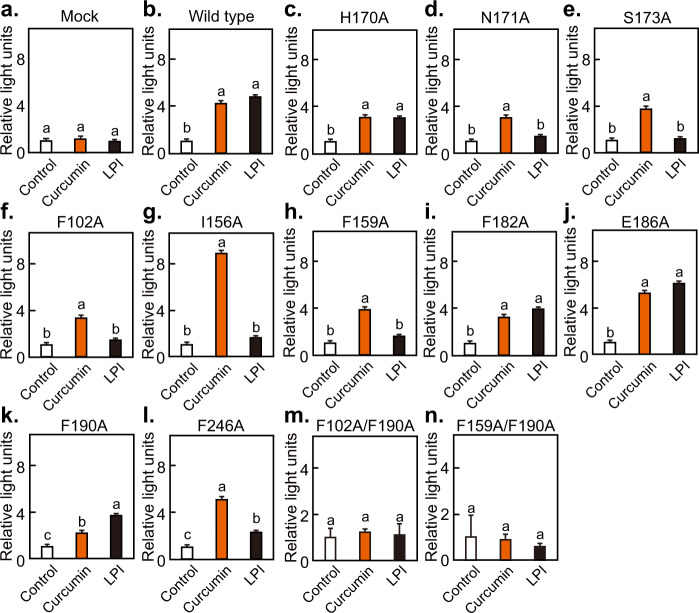
Identification of key amino acid residues in GPR55 that interact with curcumin. **a**–**n** HEK293-FT cells transfected with wild-type or mutant GPR55 expression vectors and firefly and *Renilla* luciferase reporter vectors (for serum response element (SRE)-mediated transcription) were stimulated with 10 µM curcumin or 300 nM lysophosphatidylinositol (LPI), and reporter assays were performed. Data are expressed as the mean ± s.d. (*n* = 4). Different letters indicate significant differences (*p* < 0.05).

We then constructed expression vectors of Ala mutants of amino acids predicted to interact with curcumin (hydrogen bonds: H170A^ECL2^, N171A^ECL2^, and S173A^5.31^; Van der Waals interaction: F102A^3.33^, I156A^4.60^, F159A^4.64^, F182A^5.40^, E186A^5.44^, F190A^5.47^, and F246A^6.55^). As shown in Fig. 6a–n, the luciferase reporter assay showed that the F190A^5.47^ mutation of GPR55 selectively decreased its activation by curcumin when curcumin and LPI exerted similar effects on wild-type GPR55 activation. In contrast, GPR55 mutations I156A^4.60^, N171A^ECL2^, S173A^5.31^, F102A^3.33^, F159A^4.64^, and F246A^6.55^ selectively decreased activation by LPI but not by curcumin, indicating that these residues play a lesser role in recognizing curcumin as a ligand. A unique effect was observed for the I156A^4.60^ mutant for which LPI decreased and curcumin increased activation. Subsequently, combinational mutations at important amino acid residues for van der Waals interaction with curcumin and LPI were introduced. Both double mutants with F102A^3.33^/F190A^5.47^ and F159A^4.64^/F190A^5.47^ completely abolished activation by curcumin and LPI. Overall the results indicated that F190^5.47^ of GPR55 is an important residue for its interaction with curcumin.

### Involvement of GPR55 in the biological function of curcumin

**Fig. 7:**
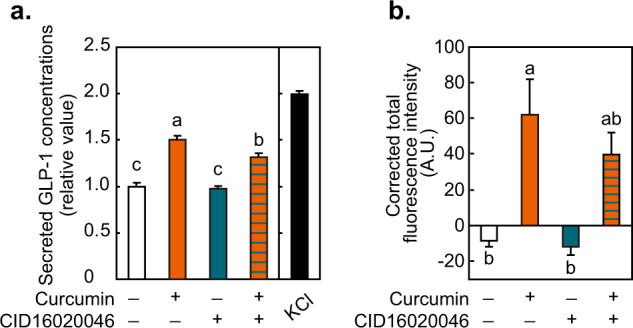
Effects of curcumin and GPR55 antagonist on GLP-1 secretion and intracellular Ca^2+^ levels in GLUTag cells. **a** GLUTag cells that had been pretreteated with CID16020046 or vehicle for 30 min were stimulated with curcumin for 1 h. KCl (50 mM) was used as positive control. Secreted GLP-1 levels in the supernatant were determined by ELISA. **b** Fura-8 fluorescence intensity was determined in the presence or absence of curcumin and CID16020046. Data are expressed as the means ± s.d. (*n* = 4). Different letters indicate significant differences (*p* < 0.05). A.U., arbitrary unit.

GPR55 is involved in GLP-1 secretion in enteroendocrine L-cells^23^, and curcumin stimulates GLP-1 secretion^7–9^. We therefore examined whether GPR55 mediates curcumin-induced GLP-1 secretion using GLUTag enteroendocrine L-cells. As shown in Fig. 7a, curcumin stimulated the secretion of GLP-1, which was inhibited by CID16020046, a GPR55 inhibitor. In addition, intracellular calcium levels were increased by curcumin, and this was attenuated in the presence of CID16020046 (Fig. 7b). These results suggest that GPR55 is involved in mediating the antiglycemic effects of curcumin.

## Discussion

To uncover the mechanisms by which food factors exert physiological functions, the identification of their cellular binding partners is a critical issue. In the present study, we screened an expression library of 258 human GPCRs and identified GPR55 as a candidate receptor for curcumin. An antagonist of GPR55 suppressed activation of GPR55 by curcumin. Furthermore, in silico binding analyses suggested that curcumin physically interacts with the ligand-binding site of GPR55. This was tested using mutant GPR55 proteins, and we identified F190^5.47^ as a key amino acid residue for GPR55 activation by curcumin. Taken together, these results strongly suggest that curcumin acts as an agonist for GPR55.

Our data indicated that curcumin bound the ligand-binding pocket of GPR55 and stimulated its activation. GPR55 belongs to the Class A family of GPCRs and a known endogenous ligand is LPI^24^. GPR55 also serves as a receptor for natural cannabinoids (e.g., Δ^9^-tetrahydrocannabinol and anandamide) and synthetic cannabinoids (e.g., CP55940 and HU210)^25,26^. Moreover, GPR55 is considered the third cannabinoid receptor, after cannabinoid receptors 1 and 2. Conversely, cannabinoid receptors 1 and 2 are not activated by LPI^27^. Ligands for GPR55 represent several different chemotypes^25^, and these analyses suggested that the ligand-binding pocket of GPR55 is relatively large. In addition, although not proven in a cell-based assays, curcumin is predicted to bind cannabinoid receptor 1 by docking simulation^28^. These results support the notion that curcumin acts as an agonist for GPR55 and suggest that curcumin may exert cannabinoid-like activity through activation of cannabinoid receptors.

Both curcumin and LPI activated GPR55-dependent transcription that was mediated by SRE- and SRF-RE but not by CRE and NFAT-RE. These results revealed that curcumin and LPI activated GPR55 coupled to Gα_12/13_ signaling, which activates Rho/Rock signaling. Our results are similar to those of previous reports that showed GPR55 being coupled to G_12/13_^29^ and G_13_^26^. Curcumin, 2,6-dimethylcurcumin, and 4′,4′′-dimethylcurcumin had comparable GPR55 activating effects, and the activation of GPR55 by curcumin and demethoxycurcumin was comparable and stronger than that by bisdemethoxycurcumin. Tetrahydrocurcumin, curcumin glucuronide, and other curcumin metabolites were inactive suggesting that the double-bonds of the central 7-carbon chain were essential for activation. Although two methoxy residues on the aromatic rings were also required for maximal activity, the addition of further methoxy residues abolished the ability to activate GPR55.

Although in silico docking approaches have advanced recently the structural information on ligand–receptor interaction, and the predictive power of the models are still insufficient. GPR55 belongs to the Class A family of GPCRs that has a consensus ligand-binding pocket^10^. Lingerfelt et al. ^30^ have identified critical amino acid residues for recognition of LPI and ML184. We have shown that residue F190^5.47^ of GPR55 that is critical for interaction with curcumin, but not with LPI. Activation of this mutant by LPI indicated that the point mutation did not alter the overall structure and function of the receptor. In addition, this site was suggested to be involved in aromatic stacks with ML184^30^, suggesting the importance of this amino acid residue for binding of certain ligands.

The induction of GPR55 activity by curcumin was observed at concentrations above 5 μM which is close to the physiologically achievable concentration. In a phase I trial, oral administration of curcumin up to 8 g/day for 3 months was found to be safe, and its plasma concentrations reached 1.77 μM^31^. After mice were orally administrated curcumin (100 mg/kg), plasma concentrations reached 2.25 μg/ml (6.1 μM)^32^, whereas the levels of curcumin in the intestine was remarkably high (177.04 μg/g)^32^. Therefore, the intestine is considered a promising target of curcumin^33^. Although low systemic bioavailability of curcumin following oral dosing is a widely recognized problem^4,34^, it is likely that curcumin reaches the concentration required to activate GPR55 in the gastrointestinal tract^4^. Curcumin stimulates the secretion of GLP-1 from gastrointestinal L-cells^7,8^ resulting in insulin secretion and a hyperglycemic effect in humans^35^. On the other hand, GPR55 is involved in glucose homeostasis^36^. GPR55 is expressed in GLUTag cells and mouse small intestine and mediates GLP-1 secretion by LPI in L-cells through activation of Rho/Rock signaling^23^, and GPR55-null mice exhibit obesity and hyperglycemia^37^. The expression of GPR55 in intestinal endocrine cells is also predicted in the human protein atlas^38^. Therefore, we have tested the role of GPR55 in mediating the physiological function of curcumin in gut L-cells. Curcumin stimulates the secretion of GLP-1 from gastrointestinal L-cells^7,8^ resulting in insulin secretion and a hyperglycemic effect in humans^35^. Our findings that curcumin stimulated GPR55 and enhanced GLP-1 secretion are consistent with the anti-hyperglycemic activity of curcumin. The GPR55 antagonist did not completely repress GLP-1 secretion by curcumin, possibly indicating a role of GPR40/120 which was also suggested as a receptor for curcumin expressed in L-cells^8^.

Curcumin affects multiple physiological processes including the maintenance of glucose homeostasis. Previous studies have shown that curcumin binds to and activates vitamin D receptor^39^, and a GPR40 antagonist suppressed the physiological function of curcumin^8^, suggesting these receptors as potential cellular targets of curcumin. Due to lack of a suitable labeled ligand, we were unable to test binding of curcumin of GPR55 directly. Nevertheless, our results strongly suggest that GPR55 functions as a potential receptor for curcumin, and that GPR55 might be involved in several physiological processes mediated by curcumin. Identification of molecular targets will advance our understanding of the molecular mechanisms by which bioactive food compounds exert their effects.

## Methods

### Materials

Curcumin and compounds **7**–**16** were prepared by chemical synthesis as described^20,40^. Bicyclopentadione isomers were prepared by autoxidation of curcumin^19^. Reduced curcumin was a mixture of tetrahydrocurcumin (70%), hexahydrocurcumin (20%) and octahydrocurcumin (10%) obtained by NaBH_4_ reduction of curcumin^22^. Compounds were used as stock solutions dissolved in DMSO.

### Cell culture

HEK293FT (Thermo Fisher Scientific, Waltham, MA, USA, RRID:CVCL_6911) and GLUTag cells (RRID:CVCL_J406)^41^ were cultured in Dulbecco’s modified Eagle’s medium (DMEM) supplemented with 10% fetal bovine serum (FBS), 100 units/mL penicillin, and 100 mg/mL streptomycin. The cells were maintained at 37 °C in an atmosphere containing 5% CO_2_ and 95% air atmosphere.

### Plasmid

To construct p4xCRE-3xSRE-TATA-Luc2P, four tandem repeats of the cAMP-response element (5′-AGCCTGACGTCAGAG-3′)^42,43^, three tandem repeats of the serum-response element (5′-AGGATGTCCATATTAGGACATCT-3′)^44,45^ derived from the human c-*fos* gene, and an adenovirus E1b TATA sequence (5′-AGGGTATATAATG-3′)^46,47^ were subsequently inserted into a pGL4.11 vector (Promega, Madison, WI, USA). Similarly, p4xCRE-TATA-Luc2P and p3xSRE-TATA-Luc2P were constructed. To construct p2xSRF-TATA-Luc2P, two tandem repeats of serum-response factor-response element (5′-TCGACTGTACTGTATGTCCATATTAGGACATCTG-3′)^48,49^ derived from the human c-*fos* gene and the TATA sequence were inserted into a pGL4.11 vector. The NFAT luciferase reporter vector was obtained from Addgene (#10959)^50^. To construct the GPCR expression vectors, we utilized the Gateway system and subcloned 258 full-length human GPCR cDNAs from pDONR201 vectors^51^ into pcDNA3.2/V5-DEST vectors (Thermo Fisher Scientific). Mutant GPR55 expression vectors were constructed by site-directed mutagenesis with Tks Gflex DNA polymerase (Takara Bio, Shiga, Japan) using primers (Supplementary Table 1).

### Expression screening and luciferase reporter assay

HEK293FT cells were seeded at a density of 8 × 10^4^ cells/well (for p4xCRE-3xSRE-TATA-Luc2P, p3xSRE-TATA-Luc2P, or p2xSRF-TATA-Luc2P) and 6 × 10^4^ cells/well (for p4xCRE-TATA-Luc2P or NFAT luciferase reporter) in 48-well plates with DMEM containing 2%FBS and 10%FBS without antibiotics, respectively. After overnight culture, cells were transfected for 24 h with GPCR expression vector, a luciferase reporter vector, and pGL4.74[*hRluc*/TK] (Promega) which were preincubated with prepared PEI MAX (Polysciences Inc., Warrington, PA, USA)^52^ and Opti-MEM (Thermo Fisher Scientific), followed by incubation with curcumin or its related compounds for 24 h (NFAT reporter) or 4 h (the other reporters). Inhibitors were added 30 min before the addition of curcumin. Soy-derived LPI (Sigma-Aldorich, St. Louis, MO, USA) was used as positive control. Luciferase reporter activity was determined as described previously^53^.

### Western blotting

HEK293FT cells were seeded at a density of ~7 × 10^5^ cells/well in a 6-well plate with DMEM containing 2% FBS. After overnight culture, cells were transfected with pcDNA3.2-GPR55-V5 using PEI MAX and Opti-MEM for 24 h, followed by incubation with curcumin for 3 h. The cells were washed with PBS(−) and sonicated in lysis buffer (20 mM Tris-HCl, pH 7.5, 150 mM NaCl, 2 mM EDTA, 1% TritonX-100, 2.5 mM sodium pyrophosphate, 1 mM β-glycerophosphate, 1 mM sodium orthovanadate, 10 μg/mL leupeptin, 1 μg/mL aprotinin, 1 mM phenylmethylsulfonyl fluoride). After centrifugation (20,000 × *g* for 10 min), the supernatant was analyzed by western blotting with monoclonal anti-V5 (SV5-Pk1, Bio-Rad, Hercules, CA, USA, RRID:AB_322378) or monoclonal anti-β-actin antibody (2D4H5, Proteintech, Rosemont, IL, USA, RRID:AB_2687938). After reaction to horseradish peroxidase-conjugated secondary antibody (Bio-Rad, RRID:AB_11125547), the immunoreactive bands were developed with Immobilon Western Chemiluminescent HRP substrate (Millipore, Bedford, MA, USA) and were detected with LAS4000 imager (GE Healthcare, Piscataway, NJ, USA). Band intensities were determined using ImageJ software version 1.53k (NIH, Bethesda, MD, USA). All blots were processed in parallel and derived from the same experiment.

### Evaluation of intracellular Ca^2+^ levels

HEK293FT cells were seeded at a density of 8 × 10^4^ cells/well in a poly-d-lysine coated 48-well plate with DMEM containing 2% FBS. After overnight culture, cells were transfected with mock or pcDNA3.2-GPR55-V5 using PEI MAX and Opti-MEM for 24 h. GLUTag cells were seeded at a density of 8 × 10^4^ cells/well in a poly-d-lysine coated 48-well plates with DMEM containing 10% FBS and incubated for 48 h. Cells were washed with standard extracellular buffer (Hepes-NaOH, pH7.5, 143.4 mM NaCl, 4.5 mM KCl, 2.6 mM CaCl_2_, 1.2 mM MgCl_2_, 5.5 mM glucose) containing 5 μM Fura-8AM (AAT Bioquest, Sunnyvale, CA, USA) for 15 min at 37 °C. After adaptation to room temperature (i.e., ~25 °C) for 15 min, the cells were washed three times and medium was replaced to fresh standard extracellular buffer containing 10 µM CID16020046 and incubated for 15 min, followed by stimulation of 10 μM (for HEK293FT cells) or 20 μM (for GLUTag cells) curcumin. Fluorescence intensity (Ex: 365 nm and Em: 530 nm) was determined with microplate reader (MTP-900, Corona Electric, Ibaraki, Japan) at 1-s intervals before and after stimulation with curcumin for 4 min. The integrated value of the intensity was calculated.

### Docking simulations

A structural model of the GPR55 in the activated state was built based on the crystal structure of the human δ-opioid receptor (Protein Data Bank [PDB] code: 4N6H)^30,54^. Its coordinates were used as the source of the GPR55 structure for in silico docking. Three-dimensional structures of curcumin (enol and diketone forms) were obtained from ZINC15, a free database of commercially available compounds for virtual screening^55^. The nonpolar hydrogen atoms of the GPR55 and curcumin molecules were merged into neighboring heavy atoms using AutoDockTools^56^. Docking simulations of each curcumin structure against the GPR55 molecule were performed ten times with different seed values using AutoDock Vina version 1.1.2^57^, generating 100 docking poses for each tautomer. The docking box was set to grids of 28 × 28 × 28 Å, which covered the extracellular binding pocket of GPR55. During the docking simulations, the rotational bands of the ligands were explicitly considered as flexible, and the receptor was treated as a rigid body. GPR55–curcumin interactions in each docking pose were analyzed using LIGPLOT^58^, and the key residues of GPR55 for curcumin binding were estimated based on how often an amino acid residue was involved in hydrogen bonding or van der Waals (vdW) interactions. This analysis procedure was automatically performed using AutoMap version 1.1.1^59^.

### Determination of secreted GLP-1 concentrations

GLUTag cells seeded at a density of 8 × 10^4^ cells/well in a 48-well plate and incubated for 48 h were washed and incubated with Krebs-Ringer bicarbonate buffer containing 0.1% bovine serum albumin and 5.5 mM glucose for 30 min. The buffer was replaced with a fresh buffer, and the cells were incubated with 10 μM CID16020046, followed by stimulation with 20 μM curcumin for 1 h. The amount of secreted GLP-1 was determined using the GLP-1 ELISA Kit Wako, High Sensitive (Wako Pure Chemical Corp.).

### Statistical analysis

Data were evaluated using Student’s *t* test or one-way analysis of variance (ANOVA) with Tukey–Kramer’s or Dunnett’s post hoc tests. Statistical analysis was performed using JMP statistical software (version 8.0.1; SAS Institute, Cary, NC, USA). Data are expressed as the mean ± standard deviation (SD), and differences were considered as statistically significant at *p* < 0.05.

### Reporting summary

Further information on research design is available in the Nature Research Reporting Summary linked to this article.

## Supplementary information

Supplemental Information

Reporting Summary

## Data Availability

The authors declare that the data supporting the findings of this study are presented within the manuscript and a supplementary file. Additional data underlying the results presented in this study are available from the corresponding author upon reasonable request.
