# Supplementary material for: Identification of G protein-coupled receptor 55 (GPR55) as a target of curcumin
Source: NPJ Sci Food. 2022 Jan 14;6:4. doi: 10.1038/s41538-021-00119-x (PMC8760322; doi:10.1038/s41538-021-00119-x)
Supplement: Supplementary file 1 — Supplemental Information [file 41538_2021_119_MOESM1_ESM.pdf]

Supplementary Information

Identification of G-protein coupled receptor 55 (GPR55) as a target of curcumin

Naoki Harada[1]*, Mai Okuyama[1], Yoshiaki Teraoka[1], Yumi Arahori[1], Yoh Shinmori[1], Hiroko Horiuchi[1], Paula B. Luis[2], Akil I. Joseph[2], Tomoya Kitakaze[1], Shigenobu Matsumura[3], Tohru Hira[4], Norio Yamamoto[5], Takashi Iuni[1], Naoki Goshima[6,7], Claus Schneider[2], Hiroshi Inui[1,8], and Ryoichi Yamaji[1].

[1]Division of Applied Life Sciences, Graduate School of Life and Environmental Sciences, Osaka Prefecture University, Sakai, Osaka 599-8531, Japan;
[2]Department of Pharmacology and the Vanderbilt Institute of Chemical Biology, Vanderbilt University Medical School, Nashville, Tennessee 37232, USA;
[3]Division of Clinical Nutrition, Graduate School of Comprehensive Rehabilitation, Osaka Prefecture University, Habikino, Osaka 583-0872, Japan;
[4]Research Faculty of Agriculture, Hokkaido University, Sapporo, Hokkaido 060-8589, Japan;
[5]R&D Planning Division, House Wellness Foods Corporation, Yotsukaido, Chiba 284-0033, Japan;
[6]Molecular Profiling Research Center for Drug Discovery, National Institute of Advanced Industrial Science and Technology, Koto-ku, Tokyo 135-0064, Japan;
[7]Department of Human Sciences, Musashino University, Koto-ku, Tokyo 135-8181, Japan;
[8]Department of Health and Nutrition, Otemae University, Osaka 540-0008, Japan.

* Corresponding author (harada@biochem.osakafu-u.ac.jp)

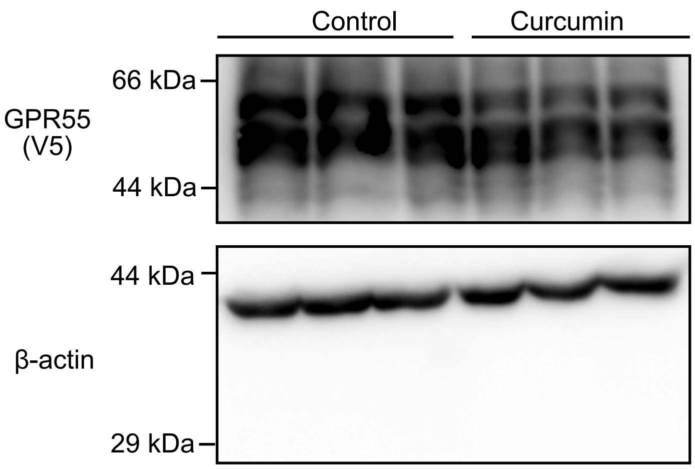

Supplementary Figure 1. Unprocessed blot images of GPR55-V5 and β-actin blots shown in Figures 1h and 1i. The experimental procedures are described in the Materials and Methods section.

Supplementary Table 1. Primers for construction of mutant GPR55 expression vectors

Primer	Sequences
Fw: GPR55/H170A	5′-CATGTGCTTCGCGAACATGTCTGATGATACCTG-3′
Rev: GPR55/H170A	5′-CAGGTATCATCAGACATGTTCGCGAAGCACATG-3′
Fw: GPR55/N171A	5′-CATGTGCTTCCACGCCATGTCTGATGATACCTG-3′
Rev: GPR55/N171A	5′-CAGGTATCATCAGACATGGCGTGGAAGCACATG-3′
Fw: GPR55/S173A	5′-GCTTCCACAACATGGCTGATGATACCTGGAGCG-3′
Rev: GPR55/S173A	5′-CGCTCCAGGTATCATCAGCCATGTTGTGGAAGC-3′
Fw: GPR55/F102A	5′-GGTGGAGTGCCTTTACGCCGTCAGCATGTACGG-3′
Rev: GPR55/F102A	5′-CCGTACATGCTGACGGCGTAAAGGCACTCCACC-3′
Fw: GPR55/I156A	5′-GACCGGAAGCATCCCTGCCTACAGTTTCCATGG-3’
Rev: GPR55/I156A	5′-CCATGGAAACTGTAGGCAGGGATGCTTCCGGTC-3′
Fw: GPR55/F159A	5′-GCATCCCTATCTACAGTGCCCATGGGAAAGTGG-3′
Rev: GPR55/F159A	5′-CCACTTTCCCATGGGCACTGTAGATAGGGATGC-3′
Fw: GPR55/F182A	5′-GGAGCGCCAAGGTCGCCTTCCCGCTGGAGGTG-3′
Rev: GPR55/F182A	5′-CACCTCCAGCGGGAAGGCGACCTTGGCGCTCC-3′
Fw: GPR55/E186A	5′-GTCTTCTTCCCGCTGGCGGTGTTTGGCTTCCTC-3′
Rev: GPR55/E186A	5′-GAGGAAGCCAAACACCGCCAGCGGGAAGAAGAC-3′
Fw: GPR55/F190A	5′-CTGGAGGTGTTTGGCGCCCTCCTTCCCATGGGC-3′
Rev: GPR55/F190A	5′-GCCCATGGGAAGGAGGGCGCCAAACACCTCCAG-3′
Fw: GPR55/F246A	5′-CCAGTCCACCTGGGGGCCTTCCTGCAGTTCCTG-3′
Rev: GPR55/F246A	5′-CAGGAACTGCAGGAAGGCCCCCAGGTGGACTGG-3′
