# Supplementary material for: Identification of G protein-coupled receptor 55 (GPR55) as a target of curcumin
Source: NPJ Sci Food. 2022 Jan 14;6:4. doi: 10.1038/s41538-021-00119-x (PMC8760322; doi:10.1038/s41538-021-00119-x)
Supplement: Supplementary file 2 — Reporting Summary [file 41538_2021_119_MOESM2_ESM.pdf]

nature portfolio

Corresponding author(s): Naoki Harada

Last updated by author(s): Oct 14, 2021

Reporting Summary

Nature Portfolio wishes to improve the reproducibility of the work that we publish. This form provides structure for consistency and transparency in reporting. For further information on Nature Portfolio policies, see our Editorial Policies and the Editorial Policy Checklist.

Statistics

For all statistical analyses, confirm that the following items are present in the figure legend, table legend, main text, or Methods section.

n/a	Confirmed	
☐	☒	The exact sample size (*n*) for each experimental group/condition, given as a discrete number and unit of measurement
☐	☒	A statement on whether measurements were taken from distinct samples or whether the same sample was measured repeatedly
☐	☒	The statistical test(s) used AND whether they are one- or two-sided<br>*Only common tests should be described solely by name; describe more complex techniques in the Methods section.*
☐	☒	A description of all covariates tested
☐	☒	A description of any assumptions or corrections, such as tests of normality and adjustment for multiple comparisons
☐	☒	A full description of the statistical parameters including central tendency (e.g. means) or other basic estimates (e.g. regression coefficient) AND variation (e.g. standard deviation) or associated estimates of uncertainty (e.g. confidence intervals)
☐	☒	For null hypothesis testing, the test statistic (e.g. *F*, *t*, *r*) with confidence intervals, effect sizes, degrees of freedom and *P* value noted<br>*Give P values as exact values whenever suitable.*
☒	☐	For Bayesian analysis, information on the choice of priors and Markov chain Monte Carlo settings
☒	☐	For hierarchical and complex designs, identification of the appropriate level for tests and full reporting of outcomes
☒	☐	Estimates of effect sizes (e.g. Cohen's *d*, Pearson's *r*), indicating how they were calculated

Our web collection on statistics for biologists contains articles on many of the points above.

Software and code

Policy information about availability of computer code

Data collection	AutoDock Vina (version 1.1.2) and AutoMap (version 1.1.1) were used in this study.
Data analysis	Band intensity was evaluated by ImageJ (version 1.53k). Statistical analysis were perfomed with JMP (version 8.0.1).

For manuscripts utilizing custom algorithms or software that are central to the research but not yet described in published literature, software must be made available to editors and reviewers. We strongly encourage code deposition in a community repository (e.g. GitHub). See the Nature Portfolio guidelines for submitting code & software for further information.

Data

Policy information about availability of data

All manuscripts must include a data availability statement. This statement should provide the following information, where applicable:

- Accession codes, unique identifiers, or web links for publicly available datasets
- A description of any restrictions on data availability
- For clinical datasets or third party data, please ensure that the statement adheres to our policy

The authors declare that the data supporting the findings of this study are presented within the manuscript and a supplementary file. Additional data underlying the results presented in this study are available from the corresponding author upon reasonable request.

Field-specific reporting

Please select the one below that is the best fit for your research. If you are not sure, read the appropriate sections before making your selection.

☒ Life sciences ☐ Behavioural & social sciences ☐ Ecological, evolutionary & environmental sciences

For a reference copy of the document with all sections, see nature.com/documents/nr-reporting-summary-flat.pdf

Life sciences study design

All studies must disclose on these points even when the disclosure is negative.

Sample size	No sample size calculation was performed. Sample sizes were chosen based on previous published works using similar methods.
Data exclusions	No sample or data were excluded.
Replication	Data were obtained from independent experiments (N=3 or 4). All findings were successfully reproduced.
Randomization	No randomization was performed.
Blinding	No blinding was performed. Data collection and analyses were done in an unbiased manner.

Reporting for specific materials, systems and methods

We require information from authors about some types of materials, experimental systems and methods used in many studies. Here, indicate whether each material, system or method listed is relevant to your study. If you are not sure if a list item applies to your research, read the appropriate section before selecting a response.

Materials & experimental systems

n/a	Involved in the study	
☐	☒	Antibodies
☐	☒	Eukaryotic cell lines
☒	☐	Palaeontology and archaeology
☒	☐	Animals and other organisms
☒	☐	Human research participants
☒	☐	Clinical data
☒	☐	Dual use research of concern

Methods

n/a	Involved in the study	
☒	☐	ChIP-seq
☒	☐	Flow cytometry
☒	☐	MRI-based neuroimaging

Antibodies

Antibodies used	Monoclonal anti-V5 (SV5-Pk1, Bio-Rad, RRID:AB_322378)<br>Monoclonal anti-β-actin (2D4H5, Proteintech, RRID:AB_2687938)<br>Goat anti-mouse IgG (H L)-HRP conjugate (Bio-Rad, RRID:AB_11125547)
Validation	All the antibodies used in this study were validated by the manufacturers for specific detection of the antigen and species reactivity and application. In addition, anti-V5 antibody was validated by comparing samples of pcDNA3.2-GPR55-V5- and mock-transfected cells.

Eukaryotic cell lines

Policy information about cell lines

Cell line source(s)	HEK293FT (RRID:CVCL_6911) was from Thermo Fisher Scientific.<br>GLUTag (RRID:CVCL_J406) was a kind gift from Dr. Daniel J. Drucker (Mount Sinai Hospital).
Authentication	No authentication was performed.
Mycoplasma contamination	Cell lines were not tested for mycoplasma contamination regularly.
Commonly misidentified lines (See ICLAC register)	No commonly misidentified cell lines were used in this study.
